# Antibacterial properties of 5-substituted derivatives of rhodanine-3-carboxyalkyl acids

**DOI:** 10.1007/s00044-017-1852-7

**Published:** 2017-03-16

**Authors:** Waldemar Tejchman, Izabela Korona-Glowniak, Anna Malm, Marek Zylewski, Piotr Suder

**Affiliations:** 1 Departament of Chemistry, Institute of Biology, Pedagogical University of Cracow, Podchorazych 2, Kraków, 30-084 Poland; 2 Department of Pharmaceutical Microbiology, Medical University of Lublin, Chodzki 1, Lublin, 20-093 Poland; 3 Jagiellonian Center of Innovation, NMR Laboratory, Bobrzyńskiego 14, Kraków, 30-348 Poland; 4 Department of Biochemistry and Neurobiology, AGH University of Science and Technology, Mickiewicza 30, Kraków, 30–059 Poland; 5 Academic Centre for Materials and Nanotechnology, AGH University of Science and Technology, Mickiewicza 30, Kraków, 30–059 Poland

**Keywords:** Rhodanine, Thiazolidine-4-one, Rhodanine-3-acetic acid, Antibacterial activity

## Abstract

A series of rhodanine 3-carboxyalkanoic acid derivatives possessing 4′-(*N,N*-dialkyl-amino or diphenylamino)-benzylidene moiety as a substituent at the C-5 position were synthesised and their antibacterial activity was screened. All the rhodanine derivatives showed bacteriostatic or bactericidal activity to the reference gram-positive bacterial strains, but lack of activity to the reference Gram-negative bacterial strains and yeast strains was observed.

## Introduction

The 2-thiazolidine-4-one derivatives traditionally named rhodanine have been known for over 100 years, and due to their fascinating properties they are still examined (Lesyk and Zimenkovsky 2004). These compounds have a broad spectrum of biological effects (Jain et al. 2012). Rhodanine derivatives show antimalarial (Kumar et al. 2007), antitubercular (Alegaon et al. 2012), cytotoxic (Chandrappa et al. 2009), antitumor (Rao et al. 2011; Lesyk et al. 2011), antiviral (Kaminskyy 2015), and antibacterial activity (Bhatti et al. 2013; Kavitha et al. 2006; Song et al. 2014).

The research to obtain new antibacterial compounds is vitally important. Recently, due to excessive and improper use of antibiotics, there has been an increasing rate of antibiotic resistance in the bacterial strains (Woodford 2003), thus new groups of compounds which may be useful as antibacterial agents have been examined. A few reports has been published regarding the rhodanine derivatives with a carboxyalkyl acid moiety at the N-3 position (Xu et al. 2012). Biological activity of hybrid compounds possessing chalcone and rhodanine-3-acetic acid has been also studied (Chen et al. 2010). Such hybrids demonstrated synergistic effect. Antibacterial activity of rhodanine derivatives and their oxygen analogues derived from 2,4-thiazolidinedione was also compared (Zvarec et al. 2012). However, the results of present study suggested that rhodanine derivatives showed greater antibacterial activity than their analogues from the 2,4-thiazolidinedione group having at the C-2 position exocyclic oxygen atom. It was shown that the activity of the rhodanine derivative correlates with the size of the substituent at the C-5 position (Pardasani et al. 2001). The research conducted by Miao et al. (2013) and Patel et al. (2013) indicated that antibacterial activity of the acid derivatives occurred when a major hydrophobic group was introduced to the arylidene substituent at the C-5 position. The best results were achieved when an aryl group additionally with an electron-withdrawing group was introduced. The rhodanine derivatives possessing a 4-(*N,N*-dimethylamino)-benzylidene substituent at the C-5 position were also examined. These compounds acted as β-lactamase inhibitors (Grant et al. 2000). Taking into account the data presented by other authors, we decided to synthesise a series of derivatives having carboxyalkyl (acetic, propionic, butyric) acid fragment at N-3 position and benzylidene para-substituent with dimethyloamino, diethylamino, dibuthyloamino or diphenylamino group at C-5 position.

### Chemistry

**Scheme 1:**
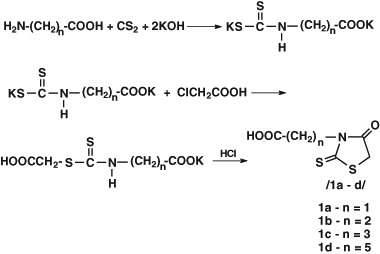
. 3-Carboxyalkylrhodanine acids synthesis

Our initial research proved that the antibacterial activity of the rhodanine derivatives which have carboxyalkyl fragment at N-3 position was more effective than the compounds with a substituent containing an amino group at C-5 position. We synthesised a series of rhodanine derivatives with a carboxyalkyl acid radical at N-3 position (acetic, propionic, butyric, caproic). The synthesis of the 3-carboxyalkylrhodanine acids (Scheme 1) was conducted according to the modified procedure proposed by Körner (1908) at the beginning of the 20th century.

**Scheme 2:**
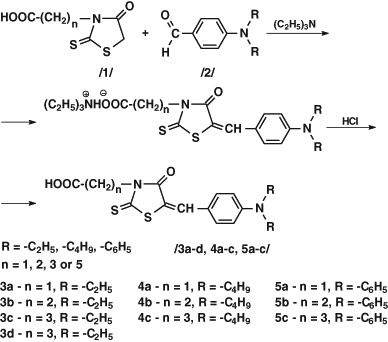
Rhodanine-3-carboxyalkyl acid condensation with aldehydes

The synthesised compounds underwent Knoevenagel condensation with 4-diethylaminobenzoic, 4-dibutylaminobenzoic aldehydes and 4-diphenylaminobenzoic aldehyde with triethylamine as a catalyst. Quaternary ammonium salts, the intermediates obtained during reactions, were not isolated but transformed to appropriate acids with hydrochloric acid (Scheme 2).

## Material and methods

All reagents for the synthesis of rhodanine derivatives were purchased from Sigma-Aldrich and used without further purification.

Melting point (uncorrected) has been determined on the Boetius apparatus. The IR spectrum has been recorded with Jasco FT IR-670 Plus spectrophotometer in the KBr disk.

The NMR spectra were obtained in CDCl_3_ on the Bruker Avance III HD spectrometer operating at 400.17 MHz (1H) and 100.62 MHz (13C) and the Varian Mercury-VX 300 spectrometer operating at 300.08 MHz (1H) and 75.46 MHz (13C), the chemical shifts (ppm) have been referenced to lock out the signal of the solvent, *J* has been expressed in Hz.

The MS analyses were obtained on the AmaZon ETD mass spectrometer (Bruker Daltonics, Bremen, Germany). Scan parameters: scan range 100–1000 m/z, positive ionisation mode. CID fragmentation were in the ion trap analyser with the aid of helium gas. The collision energy was set to ca. 1 eV. The samples were introduced into the mass spectrometer in a CH_3_OH:CHCl_3_ 1:1 solution with 0.1% HCOOH acidification.

### General procedure of rhodanine-3-alkanoic acids synthesis

The solution of 11.22 g (0.2 mol) potassium hydroxide in 50 cm^3^ of water was added to the suspension of 0.1 mol of the appropriate amino acid (aminoethanoic acid, 3-aminopropanoic acid, 4-aminobutanoic acid and 6-aminohexanoic acid). The resulting solution was cooled to 5 °C and 7.6 g (0.1 mol) carbon disulphide was added. The content of the flask was mixed at 5 °C for 7 h. The cooling bath was removed and mixing was continued in room temperature for 20 h.

The solution of 9.45 g (0.1 mol) chloroacetic acid in 50 cm^3^ water was added to the resulting solution. The solution was mixed for 7 h at the temperature below 15 °C. Next, the solution of 60 cm^3^ hydrochloric acid in 100 cm^3^ of water was added to the flask content. The resulting mixture was heated to 90 °C and kept at the temperature for 20 min. After cooling, a sediment was received, which was drained and crystallised from water.

### General procedure of rhodanine-3-alkanoic acids condensation with aldehydes

0.005 mol of appropriate rhodanine-3-alkanoic acid, 5 g molecular sieves 4 A, 25 cm^3^ isopropyl alcohol, 0.0055 mol appropriate aldehyde and 2.53 g (0.025 mol) triethylamine were placed in a flask. The mixture was heated under a reflux condenser for 5 h in nitrogen. After heating, the solution was filtered hot. The permeate was cooled and 50 cm^3^ of 2M hydrochloric acid solution was added. The resulting sediment was filtered using Büchner funnel and crystallised from isopropyl alcohol or glacial acetic acid.

#### 3a/ 5-(4′*-N,N*-diethylaminobenzylidene)-rhodanine-3-acetic acid

m.p. 239–241 °C, yield 43.48%, MS [M+1]^+^—351.1, IR cm^-3^: 1719.3C=O, 1699.9C=O conj., 1612.2, C=C exo., 1322.9 C–N, 1185.1C=S, ^1^H NMR(400 MHz, CDCl_3_+MeOD), *δ* ppm, 7.66 (s, 1H,=CH–Ar), 7.37 (d, *J* = 8.97 Hz, 2H, Ar–H), 6,70 (d, *J* = 9.00 Hz, 2H, Ar–H), 4.81 (s, 2H, HOOC–CH_2_–N), 3.43 (q, 4H, N(CH_2_CH_3_)_2_), 1.20 (t, 6H, N(CH_2_CH_3_)_2_) ^13^C NMR (101 MHz, CDCl_3_+MeOD), *δ* ppm, 12.35 (N(CH_2_ CH_3_)_2_), 44.61 (CH_2_–N), 47.70 (N(CH_2_CH_3_)_2_), 111.67 (Ar–C), 114.09 (Ar–C), 119.93 (Ar–C), 133.78 (Ar–C), 135.65 (=CH–Ar), 150.01 (S–C=CH), 167.68 (N–C=O), 168.23 (HOOC–), 193.16 (S=C–S)

#### 3b/ 5-(4′-*N,N-*diethylaminobenzylidene)-rhodanine-3-propionic acid

m.p. 202–204 °C, yield 54.70%, MS [M+1]^+^—365.1, IR cm^-3^: 1716.2C=O, 1700.9C=O conj., 1610.3, C=C exo., 1331.6 C–N, 1199.5C=S, ^1^H NMR(400 MHz, CDCl_3_), *δ* ppm, 7.69 (s, 1H,=CH–Ar), 7.40 (d, *J* = 9.00 Hz, 2H, Ar–H), 6,72 (d, *J* = 9.09 Hz, 2H, Ar–H), 4.45 (t, 2H, HOOC–CH_2_–CH_2_–N), 3.46 (q, 4H, N(CH_2_CH_3_)_2_), 2.85 (t, 2H, HOOC–CH_2_–CH_2_–N), 1.24 (t, 6H, N(CH_2_CH_3_)_2_) ^13^C NMR (101 MHz, CDCl_3_), *δ* ppm, 12.57 (N(CH_2_ CH_3_)_2_), 30.97 (CH_2_–CH_2_–N), 39.45 (CH_2_–CH_2_–N), 44.70(N(CH_2_CH_3_)_2_), 111.70 (Ar–C), 114.51 (Ar–C), 120.17 (Ar–C), 133.72 (Ar–C), 135.23 (=CH–Ar), 149.82 (S–C=CH), 167.76 (N–C=O), 175.59 (HOOC–), 192.98 (S=C–S)

#### 3c/ 5-(4′-*N,N*-diethylaminobenzylidene)-rhodanine-3-butyric acid

m.p. 155–157 °C, yield 17.06%, MS [M+1]^+^—379.1, IR cm^-3^: 1716.3C=O, 1693.2C=O conj., 1610.2C=C exo., 1340.3 C–N, 1194.7C=S, ^1^H NMR(400 MHz, CDCl_3_), *δ* ppm, 7.67 (s, 1H,=CH–Ar), 7.39 (d, *J* = 8.84 Hz, 2H, Ar–H), 6,72 (d, *J* = 9.04 Hz, 2H, Ar–H), 4.21 (t, 2H, HOOC–CH_2_–CH_2_–CH_2_–N), 3.45 (q, 4H, N(CH_2_CH_3_)_2_), 2.45 (t, 2H, HOOC–CH_2_–CH_2_–CH_2_–N), 2.09 (q, 2H, HOOC–CH_2_–CH_2_–CH_2_–N), 1.26 (t, 6H, N(CH_2_CH_3_)_2_) ^13^C NMR (101 MHz, CDCl_3_), *δ* ppm, 12.58 (N(CH_2_ CH_3_)_2_), 22.28 (CH_2_–CH_2_–CH_2_–N), 31.17 (CH_2_–CH_2_–CH_2_–N), 43.43 (CH_2_–CH_2_–CH_2_–N), 44.67 (N(CH_2_CH_3_)_2_), 111.66 (Ar–C), 114.74 (Ar–C), 120.22 (Ar–C), 133.66 (Ar–C), 134.94 (=CH–Ar), 149.75 (S–C=CH), 168.23 (N–C=O), 177.79 (HOOC–), 193.38 (S=C–S)

#### 3d/ 5-(4′*-N,N-*diethylaminobenzylidene)-rhodanine-3-caproic acid

m.p. 135–137 °C, yield 32.96%, MS [M+1]^+^—407.1, IR cm^-3^: 1717.3C=O, 1702.8C=O conj., 1615.1C=C exo., 1327.7 C–N, 1195.7C=S, ^1^H NMR(400 MHz, CDCl_3_), *δ* ppm,11.25 (br. s HOOC–) 7.66 (s, 1H,=CH–Ar), 7.39 (d, *J* = 8.97 Hz, 2H, Ar–H), 6,72 (d, *J* = 8.48 Hz, 2H, Ar–H), 4.13 (t, 2H, HOOC–CH_2_–CH_2_–CH_2_–CH_2_–CH_2_–N), 3.45 (q, 4H, N(CH_2_CH_3_)_2_), 2.39 (t, 2H, HOOC–CH_2_–CH_2_–CH_2_–CH_2_–CH_2_–N), 1.74 (m, 4H, HOOC–CH_2_–CH_2_–CH_2_–CH_2_–CH_2_–N), 1.43 (m, 2H, HOOC–CH_2_–CH_2_–CH_2_–CH_2_–CH_2_–N), 1.25 (t, 6H, N(CH_2_CH_3_)_2_) ^13^C NMR (101 MHz, CDCl_3_), *δ* ppm, 12.58 (N(CH_2_ CH_3_)_2_), 24.19 (CH_2_–CH_2_–CH_2_–CH_2_–CH_2_–N), 26.20 (CH_2_–CH_2_–CH_2_–CH_2_–CH_2_–N), 26.62 (CH_2_–CH_2_–CH_2_–CH_2_–CH_2_–N), 44.21((CH_2_–CH_2_–CH_2_–CH_2_–CH_2_–N), 44.73 (N(CH_2_CH_3_)_2_), 111.72 (Ar–C), 115.10 (Ar–C), 120.30 (Ar–C), 133.59 (Ar–C), 134.64 (=CH–Ar), 149.60 (S–C=CH), 168.14 (N–C=O), 179.47 (HOOC–), 193.33 (S=C–S)

#### 4a/ 5-(4′-*N,N*-dibutylaminobenzylidene)-rhodanine-3-acetic acid

m.p. 191–194 °C, yield 23.0%, MS [M+1]^+^—407.1, IR cm^-3^: 1716.3C=O, 1698.0C=O conj., 1636.3C=C exo., 1324.9 C–N, 1184.1C=S, ^1^H NMR(400 MHz, CDCl_3_), *δ* ppm, 7.72 (s, 1H,=CH–Ar), 7.40 (d, *J* = 8.96 Hz, 2H, Ar–H), 6,72 (d, *J* = 7.64 Hz, 2H, Ar–H), 4.95 (s, 2H, HOOC–CH_2_–N), 3.37 (t, 4H, N(CH_2_CH_2_CH_2_CH_3_)_2_), 1.63 (q, 4H, N(CH_2_CH_2_CH_2_CH_3_)_2_), 1.39 (m, 4H, N(CH_2_CH_2_CH_2_CH_3_)_2_), 0.99 (t, 6H, N(CH_2_CH_2_CH_2_CH_3_)_2_) ^13^C NMR (101 MHz, CDCl_3_), *δ* ppm, 13.92 (N(CH_2_CH_2_CH_2_ CH_3_)_2_), 20.26 (N(CH_2_CH_2_ CH_2_CH_3_)_2_), 29.32 (N(CH_2_ CH_2_CH_2_CH_3_)_2_), 44.25 (–CH_2_–N), 51.01 (N(CH_2_CH_2_CH_2_CH_3_)_2_), 112.02 (Ar–C), 114.11 (Ar–C), 120.22 (Ar–C), 133.68 (Ar–C), 135.70 (=CH–Ar), 150.17 (S–C=CH), 167.31 (N–C=O), 170.42 (HOOC–), 192.85 (S=C–S)

#### 4b/ 5-(4′-*N,N*-dibutylaminobenzylidene)-rhodanine-3-propionic acid

m.p. 163–165 °C, yield 21.99%, MS [M+1]^+^—421.2, IR cm^-3^: 1727.9C=O, 1698.0C=O conj., 1610.3C=C exo., 1336.4 C–N, 1189.9C=S, ^1^H NMR(400 MHz, CDCl_3_), *δ* ppm, 7.68 (s, 1H,=CH–Ar), 7.38 (d, *J* = 9.18 Hz, 2H, Ar–H), 6,69 (d, *J* = 8.80 Hz, 2H, Ar–H), 4.46 (t, 2H, HOOC–CH_2_–CH_2_–N), 3.37 (t, 4H, N(CH_2_CH_2_CH_2_CH_3_)_2_), 2.86 (t, 2H, HOOC–CH_2_–CH_2_–N), 1.62 (q, 4H, N(CH_2_CH_2_CH_2_CH_3_)_2_), 1.39 (q, 4H, N(CH_2_CH_2_CH_2_CH_3_)_2_), 1.02 (t, 6H, N(CH_2_CH_2_CH_2_CH_3_)_2_) ^13^C NMR (101 MHz, CDCl_3_), *δ* ppm, 13.94 (N(CH_2_CH_2_CH_2_ CH_3_)_2_), 20.27 (N(CH_2_CH_2_ CH_2_CH_3_)_2_), 29.37 (N(CH_2_ CH_2_CH_2_CH_3_)_2_), 30.95 (CH_2_–CH_2_–N), 39.45 (CH_2_–CH_2_–N), 50.87 (N(CH_2_CH_2_CH_2_CH_3_)_2_), 111.82 (Ar–C), 114.41 (Ar–C), 120.06 (Ar–C), 133.64 (Ar–C), 135.23 (=CH–Ar), 150.22 (S–C=CH), 167.77 (N–C=O), 175.49 (HOOC–), 192.96 (S=C–S)

#### 4c/ 5-(4′-*N,N-*dibutylaminobenzylidene)-rhodanine-3- butyric acid

m.p. 134–136 °C, yield 25.23%, MS [M+1]^+^—435.1, IR cm^-3^: 1710.5C=O, 1691.3C=O conj., 1637.3C=C exo., 1333.5 C–N, 1193.7C=S, ^1^H NMR(400 MHz, CDCl_3_), *δ* ppm, 11.00 (br. s, 1H, HOOC–) 7.66 (s, 1H,=CH–Ar), 7.38 (d, *J* = 8.97 Hz, 2H, Ar–H), 6.67 (d, *J* = 9.05 Hz, 2H, Ar–H), 4.21 (t, 2H, HOOC–CH_2_–CH_2_–CH_2_–N), 3.36 (t, 4H, N(CH_2_CH_2_CH_2_CH_3_)_2_), 2.45 (t, 2H, HOOC–CH_2_–CH_2_–CH_2_–N), 2.09 (t, 2H, HOOC–CH_2_–CH_2_–CH_2_–N), 1.62 (q, 4H, N(CH_2_CH_2_CH_2_CH_3_)_2_), 1.39 (m, 4H, N(CH_2_CH_2_CH_2_CH_3_)_2_), 0.99 (t, 6H, N(CH_2_CH_2_CH_2_CH_3_)_2_) ^13^C NMR (101 MHz, CDCl_3_), *δ* ppm, 13.94 (N(CH_2_CH_2_CH_2_ CH_3_)_2_), 20.27 (N(CH_2_CH_2_ CH_2_CH_3_)_2_), 22.27 (CH_2_–CH_2_–CH_2_–N), 29.38 (N(CH_2_ CH_2_CH_2_CH_3_)_2_), 31.21 (CH_2_–CH_2_–CH_2_–N), 43.43 (CH_2_–CH_2_–CH_2_–N), 50.85 (N(CH_2_CH_2_CH_2_CH_3_)_2_), 111.78 (Ar–C), 114.66 (Ar–C), 120.13 (Ar–C), 133.58 (Ar–C), 134.93 (=CH–Ar), 150.15 (S–C=CH), 168.23 (N–C=O), 178.12 (HOOC–), 193.35 (S=C–S)

#### 5a/ 55-(4′-*N,N*-diphenylaminobenzylidene)-rhodanine-3-acetic acid

m.p. 240–242 °C, yield 64.27%, MS [M+1]^+^—447.1, IR cm^-3^: 1724.1C=O, 1706.7C=O conj., 1634.4C=C exo., 1329.7 C–N, 1192.7C=S, ^1^H NMR(400 MHz, CDCl_3_), *δ* ppm, 7.76 (s, 1H,=CH–Ar), 7.53 (d, *J* = 8.96 Hz, 2H, Ar–H), 7.44–7.40 (m. 6H Ar), 7.25–7.18 (m, 6H Ar), 4.73 (s, 2H, HOOC–CH_2_–N) ^13^C NMR (101 MHz, CDCl_3_), *δ* ppm, 43.46 (–CH_2_–N), 39.87 (CH_2_–CH_2_–N), 117.67, 118.64, 119.67, 124.97, 125.85. 125.94, 125.66, 126.87, 129.05, 130.47, 130.50, 131.76, 133.32, 134.47, 166.92, (Ar–C), 145.98 (=CH–Ar), 150.65 (S–C=CH), 167.63 (N–C=O), 191.02 (HOOC–), 193.33 (S=C–S)

#### 5b/ 5-(4′-*N,N-*diphenylaminobenzylidene)-rhodanine-3-propionic acid

m.p. 212–215 °C, yield 76.09%, MS [M+1]^+^—461.1 IR cm^-3^: 1733.7C=O, 1706.7C=O conj., 1637.3C=C exo., 1338.4 C–N, 1193.7C=S, ^1^H NMR(400 MHz, CDCl_3_+MeOD), *δ* ppm, 7.61 (s, 1H,=CH–Ar), 7.32–7.23 (m. 6H Ar), 7.14–7.12 (m, 6H Ar), 6,98 (d, *J* = 8.76 Hz, 2H, Ar–H), 4.37 (t, 2H, HOOC–CH_2_–CH_2_–N), 2.71 (t, 2H, HOOC–CH_2_–CH_2_–N) ^13^C NMR (101 MHz, CDCl_3_+MeOD), *δ* ppm, 30.97 (–CH_2_–CH_2_–N), 39.87 (CH_2_–CH_2_–N), 118.24, 120.30, 124.97, 125.22, 126.05, 129.66, 132.39, 133.77 (Ar–C), 146.04 (=CH–Ar), 150.46 (S–C=CH), 167.76 (N–C=O), 172.81 (HOOC–), 192.95 (S=C–S)

#### 5c/ 5-(4′-*N,N*-diphenylaminobenzylidene)-rhodanine-3-butyric acid

m.p. 137–140 °C, yield 75.33%, MS [M+1]^+^—475.1, IR cm^-3^: 1721.2C=O, 1700.9C=O conj., 1638.23C=C exo., 1330.6 C–N, 1193.7C=S, ^1^H NMR(400 MHz, CDCl_3_), *δ* ppm, 10.90 (br. s, HOOC–), 7.67 (s, 1H,=CH–Ar), 7.37–7.33 (m, 6H Ar), 7.19–7.16 (m, 6H Ar), 7.05 (d, *J*=8.80 Hz, 2H, Ar–H), 4.23 (t, 2H, HOOC–CH_2_–CH_2_–CH_2_–N), 2.47 (t, 2H, HOOC–CH_2_–CH_2_–CH_2_–N), 2.10 (q, 2H, HOOC–CH_2_–CH_2_–CH_2_–N) ^13^C NMR (101 MHz, CDCl_3_), *δ* ppm, 22.24 (CH_2_–CH_2_–CH_2_–N), 31.10 (CH_2_–CH_2_–CH_2_–N), 43.50 (CH_2_–CH_2_–CH_2_–N), 118.50, 120.43, 124.99, 125.41, 126.09, 129.72, 132.40, 133.58 (Ar–C), 146.14 (=CH–Ar), 150.40 (S–C=CH), 168.12 (N–C=O), 177.75 (HOOC–), 193.27 (S=C–S)

### Antibacterial activity assay in vitro

The 5-substituted derivatives of rhodanine-3-carboxyalkyl acids were screened for antibacterial and antifungal activities by micro-dilution broth method using Mueller-Hinton broth and Mueller-Hinton broth with 5% lysed sheep blood for growth of non-fastidious and fastidious bacteria, respectively or Mueller-Hinton broth with 2% glucose for growth of fungi. Minimal inhibitory concentration (MIC) of the tested derivatives were evaluated for the panel of the reference microorganisms from American Type Culture Collection (ATCC), including Gram-negative bacteria (*Escherichia coli* ATCC 25922, *Salmonella typhimurium* ATCC14028, *Klebsiella pneumoniae* ATCC 13883, *Pseudomonas aeruginosa* ATCC 9027, *Proteus mirabilis* ATCC 12453), gram-positive bacteria (*Staphylococcus aureus* ATCC 25923, *Staphylococcus aureus* ATCC 6538, *Staphylococcus epidermidis* ATCC 12228, *Micrococcus luteus* ATCC 10240, *Bacillus subtilis* ATCC 6633, *Bacillus cereus* ATCC 10876*, Streptococcus pyogenes* ATCC 19615, *Streptococcus pneumoniae* ATCC 49619, *Streptococcus mutans* ATCC 25175), and fungi (*Candida albicans* ATCC 10231, *Candida parapsilosis* ATCC 22019).

The 5-substituted derivatives of rhodanine-3-carboxyalkyl acids dissolved in dimethylosulfoxide (DMSO), were first diluted to the concentration (1000 µg/mL) in an appropriate broth medium recommended for bacteria or yeasts. Then, using the same media, serial two-fold dilutions were made in order to obtain final concentrations of the tested derivatives ranged from 0.98 to 1000 µg/mL. The sterile 96-well polystyrene microtitrate plates (Nunc, Denmark) were prepared by dispensing 200 µl of appropriate dilution of the tested derivatives in broth medium per well. The inocula were prepared with fresh microbial cultures in sterile 0.85% NaCl to match the turbidity of 0.5 McFarland standard and 2 μl were added to wells to obtain final density of 1.5 × 10^6^ CFU/ml for bacteria and 5 × 10^4^ CFU/ml for yeasts; CFU—colony forming units. After incubation (bacterial strains—35 °C for 24 h, yeast strains—30 °C for 48 h), the MICs were assessed visually as the lowest concentration of the 5-substituted derivatives of rhodanine-3-carboxyalkyl acids showing complete growth inhibition of the reference microbial strains. Appropriate DMSO control (at a final concentration of 10%), a positive control (containing inoculum without the tested derivatives) and negative control (containing the tested derivatives without inoculum) were included on each microplate.

Minimal bactericidal concentration (MBC) or minimal fungicidal concentration (MFC) was determined by subculturing 100 μl of the microbial culture from each well that showed through growth inhibition, from the last positive one and from the growth control onto the recommended agar plates. The plates were incubated at 35 °C for 24 h and the MBC/MFC was defined as the lowest concentration of the 5-substituted rhodanine-3-carboxyalkyl acids without growth of microorganisms. Ciprofloxacin and vancomycin were used as the standard drugs (Table 1). Each experiment was repeated in triplicate. Representative data is presented.

**Table 1:** MIC (µg/mL), MBC (µg/mL) of vancomicin and ciprofloxacin towards reference Gram-positive bacterial strains

Microorganisms	Vancomicin			Ciprofloxacin		
	MIC (µg/mL)	MBC (µg/mL)	MBC/MIC ratio	MIC (µg/mL)	MBC (µg/mL)	MBC/MIC ratio
*S. aureus* ATCC6538	0.49	1.95	4	0.24	0.24	1
*S. aureus* ATCC25923	0.98	7.81	8	0.49	0.49	1
*S. epidermidis* ATCC12228	0.98	0.98	1	0.49	0.49	1
*M. luteus* ATCC10240	0.12	0.12	1	0.98	1.95	2
*B. subtilis* ATCC6633	0.24	0.49	2	0.03	0.12	4
*B. cereus* ATCC10876	0.98	15.6	16	0.12	0.12	1
*S. pyogenes* ATCC19615	0.24	0.49	2	–	–	–
*S. pneumoniae* ATCC49619	0.24	0.49	2	–	–	–
*S. mutans* ATCC25175	0.98	0.98	1	–	–	–

## Results and discussion

### Chemistry

All the resulting 3-carboxyalkanoic acid derivatives occurred as crystalline solids red in colour. They were characterised by high solubility in polar solvents (alcohols, glacial acetic acid).

The comparison of the condensation reaction yield of all three groups of the compounds /**3a-d**/, /**4a-c**/ and /**5a-c**/ indicated that 4-*N,N*-diphenylaminobenzoic aldehyde had the highest activity in condensation reactions among the aldehydes used. 4-dibutylaminobenzoic aldehyde was characterised by the lowest activity.

The characteristic bands deriving from the stretching C=O and C=S groups vibrations were present in the IR spectra of all the researched compounds. The C=O group vibrations ranged from 1727.9 to 1710.5 cm^-1^, whereas C=S group vibrations ranged from 1195.7 to 1184.1 cm^-1^.

The MS spectra were very simple. The highest intensity had always the [M+1]^+^ ion peak. In most cases it reached 100%.

The ^1^H NMR spectra contained a very characteristic signal deriving from the proton in =CH–Ar unit. It was a singlet, which was present in the 7.61–7.76 ppm range of chemical shifts. Position of the signal from a methine proton in this range showed that the condensation reaction carried out to Z isomers (Hardej et al. 2010). The ^13^C NMR spectra were characterised by the signal from the carbon atom bound with exocyclic sulphur atom. It was present in the 192.85–193.38 ppm range of chemical shifts.

### Antibacterial activity

The antimicrobial activity of rhodanines has been known for over 50 years. The design and synthesis of antibacterial agents based on this heterocycle have been reported in numerous studies (Pardasani et al. 2001; Grant et al. 2000; Gandhe and Gautam 2004; Tomasic and Peterlin Masic 2012). The 5-ylidene-4-thiazolidinones and 4-thiazolidinone-3-carboxylic acids are the most studied and promising 4-thiazolidinones in the context of creating new drug-like molecules (Lesyk and Zimenkovsky 2004; Lesyk et al. 2011). It was shown that introduction of substituents (mainly those containing a carboxyl group) in position N3 is the chemical path to the design of new compounds with a significant biological activity and decreased toxicity (Bhat et al. 2004). In present study, the antimicrobial assay of the novel 5-substituted derivatives of rhodanine-3-carboxyalkyl acids was carried out towards reference strains using a serial dilution method to obtain the MIC. None of the tested derivatives had activity against gram-negative bacteria (*Escherichia coli* ATCC25922, *Salmonella typhimurium* ATCC14028, *Klebsiella pneumoniae* ATCC13883, *Pseudomonas aeruginosa* ATCC9027, *Proteus mirabilis* ATCC12453), and yeasts (*Candida albicans* ATCC10231, *Candida parapsilosis* ATCC22019) (MIC > 1000 µg/mL, data not shown). Tables 2–4 summarised the results obtained for the MICs of the 10 target compounds (**3a**–**d, 4a**–**c, 5a**–**c**) to the gram-positive bacteria: staphylococi (*Staphylococcus aureus* ATCC25923, *Staphylococcus aureus* ATCC6538, *Staphylococcus epidermidis* ATCC12228); micrococci (*Micrococcus luteus* ATCC10240), bacilli (*Bacillus subtilis* ATCC6633, *Bacillus cereus* ATCC10876) and streptococci (*Streptococcus pyogenes* ATCC19615, *Streptococcus pneumoniae* ATCC49619, *Streptococcus mutans* ATCC25175). Ciprofloxacin and vancomycin were used as positive controls (Table 1). Mild to moderate activity (MIC 125–1000 µg/mL) of the all synthesised derivatives was observed towards streptococci. The new rhodanine compounds showed different activity from moderate to very strong against other tested gram-positive bacteria, i.e., staphylococci, micrococci, and bacilli, depending on the strain and the synthesised compound. The first group of derivatives /**3a**–**d**/ was less active towards the tested gram-positive strains (MIC 15.6–250 µg/mL) as compared to the second group /**4a**–**c**/ of derivatives (MIC 1.95–7.8 µg/mL) and the third group /**5a**–**c**/ of derivatives (MIC 1.95–15.6 µg/mL). The most active compounds were /**5a**/ and /**5b**/ showing very strong bioactivity with MIC 1.95 µg/mL. The low values of MBC/MIC ratio (2–4) for /**5a**/ suggested its bactericidal power in contrast to higher values (8–16) for /**5b**/ indicating bacteriostatic activities except for bactericidal activity of /**5b**/ against *B. subtilis* ATCC 6633 (MBC/MIC 1). The remaining derivatives showed bactericidal (MBC/MIC ≤ 4) or bacteriostatic activity against the tested bacteria (MBC/MIC > 4), depending on the strain and the rhodanine compound.

**Table 2:** MIC (µg/mL), MBC (µg/mL) of **3a, 3b, 3c**, and **3d** derivatives towards reference gram-positive bacterial strains

Microorganisms	**3a**			**3b**			**3c**			**3d**		
	MIC (µg/mL)	MBC (µg/mL)	MBC/MIC ratio	MIC (µg/mL)	MBC (µg/mL)	MBC/MIC ratio	MIC (µg/mL)	MBC (µg/mL)	MBC/MIC ratio	MIC (µg/mL)	MBC (µg/mL)	MBC/MIC ratio
*S. aureus* ATCC6538	250	1000	4	31.25	>1000	>32	62.5	1000	16	31.25	>1000	>32
*S. aureus* ATCC25923	125	>1000	>8	31.25	1000	32	31.25	>1000	>32	15.6	>1000	>64
*S. epidermidis* ATCC12228	125	1000	8	31.25	>1000	>32	31.25	1000	32	31.25	>1000	>32
*M. luteus* ATCC10240	125	>1000	>8	15.6	62.5	4	62.5	>1000	>16	15.6	>1000	>64
*B. subtilis* ATCC6633	62.5	250	4	15.6	>1000	>64	15.6	62.5	4	7.8	500	64
*B. cereus* ATCC10876	62.5	>1000	16	125	>1000	>4	15.6	>1000	>64	7.8	>1000	>128
*S. pyogenes* ATCC19615	250	>1000	>4	125	125	1	125	>1000	>4	250	500	2
*S. pneumoniae* ATCC49619	125	250	2	500	>1000	>2	125	500	4	250	500	2
*S. mutans* ATCC25175	250	>1000	>4	500	>1000	>2	500	>1000	>2	1000	>1000	Nd

**Table 3:** MIC (µg/mL), MBC (µg/mL) of **4a, 4b**, and **4c** derivatives towards reference gram-positive bacterial strains

Microorganisms	**4a**			**4b**			**4c**		
	MIC (µg/mL)	MBC (µg/mL)	MBC/MIC ratio	MIC (µg/mL)	MBC (µg/mL)	MBC/MIC ratio	MIC (µg/mL)	MBC (µg/mL)	MBC/MIC ratio
*S. aureus* ATCC6538	7.8	15.6	2	3.9	>1000	>256	3.9	>1000	>256
*S. aureus* ATCC25923	3.9	62.5	16	3.9	>1000	>256	3.9	>1000	>256
*S. epidermidis* ATCC12228	3.9	62.5	16	3.9	7.8	2	3.9	>1000	>256
*M. luteus* ATCC10240	3.9	125	32	3.9	7.8	2	3.9	>1000	>256
*B. subtilis* ATCC6633	3.9	3.9	1	1.95	3.9	2	1.95	3.9	2
*B. cereus* ATCC10876	3.9	125	32	3.9	3.9	1	3.9	3.9	1
*S. pyogenes* ATCC19615	125	>1000	>8	125	500	4	500	>1000	Nd
*S. pneumoniae* ATCC49619	125	500	4	125	500	4	500	1000	2
*S. mutans* ATCC25175	>1000	>1000	Nd	1000	>1000	Nd	1000	>1000	Nd

**Table 4:** MIC (µg/mL), MBC (µg/mL) of **5a, 5b**, and **5c** derivatives towards reference gram-positive bacterial strains

Microorganisms	**5a**			**5b**			**5c**		
	MIC (µg/mL)	MBC (µg/mL)	MBC/MIC ratio	MIC (µg/mL)	MBC (µg/mL)	MBC/MICratio	MIC (µg/mL)	MBC (µg/mL)	MBC/MIC ratio
*S. aureus* ATCC6538	1.95	7.8	4	1.95	31.5	16	15.6	>1000	>64
*S. aureus* ATCC25923	1.95	7.8	4	1.95	31.5	16	1.95	125	64
*S. epidermidis* ATCC12228	1.95	7.8	4	1.95	31.5	16	1.95	125	64
*M. luteus* ATCC10240	1.95	7.8	4	1.95	15.6	16	3.9	125	32
*B. subtilis* ATCC6633	1.95	3.9	2	1.95	1.95	1	1.95	1.95	1
*B. cereus* ATCC10876	1.95	7.8	4	1.95	15.6	8	3.9	62.5	16
*S. pyogenes* ATCC19615	125	1000	4	125	>1000	>8	125	>1000	>8
*S. pneumoniae* ATCC49619	250	500	2	125	500	4	125	500	4
*S. mutans* ATCC25175	500	>1000	Nd	>1000	>1000	Nd	>1000	>1000	Nd

In the present study, most of synthesised compounds (**4a**–**c** and **5a**–**c**) exhibited strong antibacterial activity amongst the tested gram-positive bacteria, although the mechanism of action is not yet clearly understood. However, rhodanines seem to be inhibitors of the bacterial enzyme MurB (Andres et al. 2000). The enzyme MurB, an NADPH dependant enolpyruvyl reductase, is responsible for the second committed step of bacterial peptidoglycan biosynthesis; it means that rhodanines could be expected to be bactericidal. Peptydoglycan is an essential component of the cell wall of both Gram-positive and Gram-negative bacteria and enzyme MurB is found in both of them. It would be expected that rhodanines might possess a broad spectrum of antibacterial activity. The differences in biological activity of rhodanines to gram-positive and gram–negative bacteria could be explained by the differences in their cell wall structure and thus in the permeability. Peptydoglycan is major component (90%) of the gram-positive cell wall, whereas in Gram-negative bacteria, peptydoglycan, constituting 10% of cell wall, lies between cytoplasmic membrane and the outer lipid byliayer containing lipopolysaccharide, porins, adhesins which create additional barrier to cross by.

In this study, the preliminary remarks of the structure activity dependence can be noted. Comparison the MIC values determined for the newly synthesised rhodanine derivatives allowed to state that the basic factor increasing the activity to prevent bacteria growth is the size of the substituent at the C-5 position. The number of the carbon atoms present in the connector between carboxylic group and the 2,4-thiazolidinedione core is of much less importance. The influence of the connector length on the activity to suppress bacterial growth is noticeable when 5 atoms of carbon are present in the connector. The derivatives which have an acetic, propionic and butyric acid fragment at N-3 position and have the same substituent at C-5 position, demonstrated similar ability to suppress the growth of Gram-positive bacteria. In many cases the activity was identical.

Determining the MBC value allowed to establish the activity to kill bacteria or inhibit its growth. Antimicrobial agent are usually regarded as bactericidal if MBC value is higher no more than four times the MIC value (French 2006).

It was established that /**5a**/ and /**4b**/ 5-(4′-dibutylaminobenzylidene)-4-oxo-2-thioxo-3-thiazolidine acetic acid, as well as 5-(4′-diphenylaminobenzylidene)-4-oxo-2-thioxo-3-thiazolidine acetic acid and 5-(4′-diphenylaminobenzylidene)-4-oxo-2-thioxo-3-thiazolidine propionic acid had antibacterial effect on the majority of the gram-positive bacteria strains.

It was surprising that increasing the number of carbon atoms in the connector resulted in decreased antibacterial activity of 5-(4′-diphenylaminobenzylidene)-4-oxo-2-thioxo-3-thiazolidine butyric acid.

## Conclusions

A series of new rhodanine-3-carboxyalkyl acid derivatives possessing *p*-*N,N*-benzylidenedialkylamine moieties and *p*-*N,N*-bezylidenediphenylamine as a substituent at the C-5 were synthesised. The compounds were characterised by antibacterial activity on the tested Gram-positive strains of bacteria, however without biological activity on gram-negative bacteria and yeasts.

It was observed that there was a dependency between the growth of the substituent size at C-5 position of the rhodanine ring and the antifungal activity growth. The derivatives having a *p*-*N,N*-bezylidenediphenylamine fragment at the C-5 position were characterised by the highest antibacterial activity. The increase of activity was probably caused by higher hydrophobicity of the aryl groups in comparison to the alkyl groups, which has been suggested by previous research (Miao et al. 2013; Patel et al. 2013). It was also established that the size of the connector between the carboxylic group and rhodanine ring had a limited influence on the antibacterial activity. The results have indicated the future direction of the research aiming at synthesis of the compounds characterised by higher antibacterial activity.
